# “You knew you had to be there, it had to be done”: Experiences of health professionals who faced the COVID-19 pandemic in one public hospital in Spain

**DOI:** 10.3389/fpubh.2023.1089565

**Published:** 2023-04-20

**Authors:** María Nieves Rodríguez-Madrid, Guadalupe Pastor-Moreno, Enrique Albert-Lopez, María Pastor-Valero

**Affiliations:** ^1^Programa de Doctorado en Ciencias de la Salud, Universidad de Sevilla, Seville, Spain; ^2^Escuela Andaluza de Salud Pública, Granada, Spain; ^3^Grupo 50 del CIBER en Epidemiología y Salud Pública (CIBERESP), Instituto de Salud Carlos III, Ministerio de Ciencia e Innovación del Gobierno de España, Madrid, Spain; ^4^Instituto de Investigación Biosanitaria ibs.GRANADA, Granada, Spain; ^5^Departamento de Medicina Interna, Hospital Universitario de Basurto, Bilbao, Spain; ^6^Departamento de Salud Pública, Historia de la Ciencia y Ginecología, Universidad Miguel Hernández de Elche, Sant Joan d'Alacant, Spain; ^7^Grupo 26 del CIBER en Epidemiología y Salud Pública (CIBERESP), Instituto de Salud Carlos III, Ministerio de Ciencia e Innovación del Gobierno de España, Madrid, Spain; ^8^Programa de pós-graduação em Saúde Coletiva, Departamento de Medicina Preventiva, Faculdade de Medicina FMUSP, Universidade de São Paulo, Sao Paulo, Spain

**Keywords:** COVID-19 pandemic, health professionals, contingency plan, organizational changes, quality of care provision, coping strategies, qualitative research

## Abstract

**Introduction:**

The COVID-19 pandemic highlighted the lack of a government contingency plan for an effective response to an unexpected health crisis. This study uses a phenomenological approach to explore the experience of healthcare professionals during the first three waves of the COVID-19 pandemic in a public health hospital in the Valencia region, Spain. It assesses the impact on their health, coping strategies, institutional support, organizational changes, quality of care, and lessons learned.

**Methods:**

We carried out a qualitative study with semi-structured interviews with doctors and nurses from the Preventive Medicine, Emergency, and Internal Medicine Services and the Intensive Care Unit, using the Colaizzi’s 7-step data analysis method.

**Results:**

During the first wave, lack of information and leadership led to feelings of uncertainty, fear of infection, and transmission to family members. Continuous organizational changes and lack of material and human resources brought limited results. The lack of space to accommodate patients, along with insufficient training in treating critical patients, and the frequent moving around of healthcare workers, reduced the quality of care. Despite the high levels of emotional stress reported, no sick leave was taken; the high levels of commitment and professional vocation helped in adapting to the intense work rhythms. Healthcare professionals in the medical services and support units reported higher levels of stress, and a greater sense of neglect by their institution than their colleagues in managerial roles. Family, social support, and camaraderie at work were effective coping strategies. Health professionals showed a strong collective spirit and sense of solidarity. This helped them cope with the additional stress and workload that accompanied the pandemic.

**Conclusion:**

In the wake of this experience, they highlight the need for a contingency plan adapted to each organizational context. Such a plan should include psychological counseling and continuous training in critical patient care. Above all, it needs to take advantage of the hard-won knowledge born of the COVID-19 pandemic.

## 1. Introduction

On 30 January, the WHO declared a COVID epidemic—caused by SARS-CoV-2 (1). Since then, the pandemic has had an unstable evolution, with peaks and troughs in cumulative incidence that have varied by country, and by region within individual countries. In Spain, the National Epidemiological Monitoring Network (Red Nacional de Vigilancia Epidemiológica) defined the first wave: from January to 21 June 2020; the second wave: from 22 June to 6 December 2020, and the third wave: from 7 December 2020 to 14 March 2021 (2). The onset of the pandemic highlighted the lack of contingency planning by governments and the health system. In most countries, prevention and tracking systems failed, there was a shortage of personal protective equipment (PPE), respirators, hospital beds, knowledge of diagnostic tests, and drug treatment (3, 4). Spain lacked a national plan to respond effectively to this health crisis, to address not only the organizational changes required at all levels of care, but also such aspects as leadership and links between organizations (5, 6). Results from several reviews of the experiences of healthcare workers coping with the COVID-19 pandemic show that front-line staff, especially nurses, and staff with little work experience, were at higher risk of anxiety, depression, stress, and insomnia (7–10). Also, factors such as female gender, level of responsibility within the service, and whether the hospital was in a severely affected area also played a role (8). Similarly, factors that reduced the impact were social and psychological support and regular physical exercise (9). A review of qualitative studies (11) identified factors that affected the experience of healthcare workers and their support needs during the pandemic using a comprehensive model that assesses the interaction of individual, interpersonal, institutional, social, and political domains. There are few studies analyzing the interaction of these different domains and comparing the experience of healthcare workers in different hospital services, with different responsibilities and roles (face-to-face care, management) during the pandemic.

The present study was carried out in response to the following objectives: (1) to explore the experiences of healthcare workers from different hospital services who worked during the first three waves of the COVID-19 pandemic in a hospital in the public network of the Valencian Community; (2) To assess the impact of the work and the support received on the quality of care and the health of these healthcare workers; (3) To examine the coping strategies that developed; and (4) To identify proposals for improvement in management, quality of care, and quality of work in the face of a pandemic. In the geographical area covered by our study, the first wave did not have a major impact on the volume of patients. In fact, all interviewees agreed that it was a period of preparation for future waves. The third wave is described as having the greatest impact on work overload due to the number of patients and the emotional strain experienced by the vast majority of the healthcare professionals. Indeed, in the Valencian Community, the third wave was the most severe, with death rates per 100,000 inhabitants of 51.17 compared to 17.65 in the Community of Madrid autonomous region (12). Moreover, it was the only region of Spain where the third wave exceeded the previous two in deaths (13).

## 2. Materials and methods

### 2.1. Design

This is a qualitative study with a phenomenological approach using a semi-structured interview technique. The phenomenological approach employs modes of discourse that try to merge cognitive and non-cognitive factors. By these terms, we mean that not only do we understand things intellectually or conceptually, but we also experience things in corporeal, relational, enactive, and situational modalities (14). This study follows the principles of qualitative research and used the COnsolidated criteria for REporting Qualitative research (COREQ) (15).

### 2.2. Participants, scope of study, and recruitment

The study population consisted of healthcare professionals: doctors, nurses, and auxiliary nursing care technicians (ANCs) from a public university hospital in the Valencian Community (Spain), part of the National Health System (Sistema Nacional de Salud—SNS), which serves a population of more than 200,000 people. Participants had to meet the following inclusion criteria: (1) to have been working during the first three waves of the COVID-19 pandemic in one of the following services or units: the Preventive Medicine, Emergency, and Internal Medicine Services and the Intensive Care Unit. For a more heterogeneous discourse, it included an equal number of male and female participants, having different ranks and roles (heads/supervisors/management, senior doctors, junior doctors, and residents). Participants were recruited by means of purposive sampling using the snowball technique between 31 May and 15 July 2021.

A heterogeneous sample of 14 healthcare professionals was recruited, of diverse demographic characteristics in terms of age, gender, functions performed, and years of experience.

### 2.3. Data collection

A script for conducting the semi-structured interview was developed based on the literature review, the results of a previous study, and consultation with experts (Supplementary Appendix 1). Participants were selected and contacted by telephone through key informants. For those who accepted to participate in the study, their personal email address was requested. Next, an interviewer trained in semi-structured interviews sent them an email and offered detailed information about the project, answering doubts and questions. After obtaining informed consent, a date was set for the interview, which was conducted through video conference using the google meet platform, outside hospital working hours. The interviews lasted 43 min on average and were recorded in their entirety with prior authorization having been obtained.

### 2.4. Analytical procedure

The Colaizzi’s 7-step method was used for data analysis (16, 17). The first three steps were: verbatim transcription of the interviews; verbatim reading of the texts; and annotations based on analytical intuitions. The fourth step consisted of the disclosure analysis using the NVivo 12 program (18), classifying the information into the categories and subcategories of nodal analysis set out in Table 1. These were established before starting the classification, based on the questions in the questionnaire. This framework obtains for each of the different sections of this study. Briefly, the discourse analysis took into account the interpretations of the discursive positions according to the characteristics of the participants. In this sense, the discourses were segmented whenever possible by: type of service, work role, and function performed. The focus was on perceptions, feelings/sensations, excuses, explanations, and/or justifications. The initial premise was based on prior knowledge, linguistic resources, points of view, silences, contradictions, and opinions, whether shared or not with the reference group of which they were part. The symbolic configurations and semantic spaces within and between the different texts were also analyzed. The fifth step included the description of the phenomenon, integrating all the resulting ideas. This was achieved by combining all the theme clusters, emergent themes and formulated meanings into a description to create an overall structure. The next step (six) described the fundamental structure of the phenomenon, with a synthesis of the main findings according to the objectives. The analysis was performed independently and triangulated between the interviewer and another member of the research team, followed by discussion, and consensus as a procedure for validation and quality control of the results obtained. Finally, in the 7-step, results were returned to the participants to validate the findings.

**Table 1:** Analysis of categories.

Categories	Subcategories	Headings
Experiences, feelings, initial concerns	First reactions	1. How did they react on hearing the news: initial feelings and concerns
	Initial feelings	
	Higher concerns	
	Increased impact	
Management of the pandemic in the first and subsequent waves	Changes in usual work performance	2. Organizational changes to the services during the first and successive waves
	Coordination with other services.	
Impacts	On the provision of care	3. Impacts of the pandemic on care provision
	On the health of health professionals	4. Impacts of the pandemic on the health of healthcare professionals.
Aid and support	At the institutional level: Facilitating factors and inhibitors of the level of emotional overload.	5. Institutional sources of support, coping strategies, and feelings of support
	At the personal level: Coping strategies, Feelings of support,	
Assessment of the experience	Level of personal satisfaction (success, challenge, failure…)	6. Recovery level
	Organizational aspects that have, improved, worsened, remain the same Positive and negative organizational aspects	7. Proposals for improvement
Other emergent themes		

### 2.5. Ethical aspects

The present study followed the guidelines set out by the Declaration of Helsinki and conformed to the principles of medical research ethics. Prior to interview, all participants signed an informed consent form. Their participation was voluntary and anonymous, and they could withdraw from the study at any time without explanation. During the interview, the privacy and comfort of the participants was a priority at all times, as was confidentiality. The research was approved by the ethical committee of the hospital. However, due to a confidentiality agreement between the participants and the research team, the name of the hospital cannot be disclosed.

### 2.6. Rigor

The results of the analysis were presented to the participants, obtaining their agreement, with both parties deciding to remove identifiable information from the verbatim quotes that feature in this article.

## 3. Results

### 3.1. Study sample

The sample constitutes 14 health professionals: eight women and six men, with professional experience ranging between 4 and 22 years, and included a Family Medicine doctor who moved from the health center to work in the hospital’s Emergency Service at the start of the pandemic. Some healthcare professionals who usually carried out healthcare tasks were involved in management tasks (organization of human and material resources, updating of action protocols) and others, with exclusive usual management tasks were involved in patient care or search for effective treatments, working, at the same time, both tasks. Table 2 describes the detailed characteristics of the healthcare professionals who participated in the study.

**Table 2:** Job profile characteristics of the participants.

Service	Male	Female	Job title	Role	Cod_ Number **
Preventive medicine		X	Nursing professional (hospital)	Management	3
		X	Head of service/section	Management	5
Emergency Service		X	Head of service/section	Management	10
	X		Physician	Patient care	13
	X		Nursing professional (hospital)	Management	8
Internal Medicine Service, Infectious Diseases Unit (IDU)	X		Nursing professional (HHU*)	Management	2
		X	Nursing professional (HHU)	Patient care	9
		X	Physician	Patient care	4
	X		Physician	Department Head	6
Intensive care unit (ICU)	X		Physician	Management	7
		X	Nursing professional (hospital)	Patient care and management	12
		X	Nursing professional (hospital)	Patient care	14
	X		Nursing professional (hospital)	Patient care	1
		X	Physician	Patient care	11

*Home Hospitalization Unit (HHU), modality of healthcare focused on providing specialized hospital care to patients at home. **Identification’s number of each participant, assigned according to the date of the interview.

### 3.2. Initial experiences, feelings, and concerns: How the news was received

In general, the healthcare professionals in the services consulted responded to the news of the epidemic unrealistically and inconsistently. In the Internal Medicine Service, the news was received as simply another health alert, and in Preventive Medicine with skepticism and disbelief, while in Emergency Services and the Intensive Care Unit it was perceived as an opportunity for recognition of their specialty.

“…one person told me: have you heard? It looks like it’s pneumonia, … and I said to myself that it was just a routine case, wasn’t it? And, towards the end of January, that’s when we started to get scared. I remember we had a meeting … we had a meeting between the IDU and the Emergency Services, the management. And in that meeting I remember (laughs) saying that the risk was low, that we were going to have one or no cases in our country …! That’s how it all started.”

“For me, in terms of work, it was good because thanks to the pandemic I had a contract as a normal person [better]. There was a lot of expectation …, it was the first time I’d heard so much talk about my specialty, about my work, […] I had a feeling of contentment, of feeling useful in a pandemic …”

In the first wave, the combination of lack of leadership and of information led to feelings of helplessness and uncertainty due to the impossibility of carrying out adequate planning. This was most evident in Emergency, and Internal Medicine Services, and ICU.

“… fundamentally, the problem in the first wave was the uncertainty, that nobody was taking the reins. Many times you knew what you had to do, but it wasn't contemplated in the guidelines, you knew what you had to do, but you didn’t have the means to do it.”

All healthcare professionals involved in direct patient care repeatedly expressed feelings of fear of infection and of transmission to household members and/or at-risk family members, aggravated by the lack of protective equipment.

“… we lacked material …, the first month and a half was horrible because we knew we were going into battle, that we would be coming under fire, and we didn’t have bulletproof vests.”

Healthcare professionals in coordination and management roles expressed fear over safety conditions at work and of reactions from colleagues and/or workers’ representatives at having to implement guidelines that were unfeasible given the lack of equipment.

“In the first wave, I was very afraid, with the whole equipment issue, … everything was uncertain and the daily fear of being left without equipment. And the pressure from other groups …, from the nursing staff, the trade union, we needed answers, and they weren’t coming. But, of course, the guidelines said: you need this. But there’s nothing left!”

### 3.3. Organizational changes in the services during the first and subsequent waves

The first task of the Preventive Medicine Service was to manage and continuously update the operational guidelines of the other services. A technical commission was set up for the coordination of the pandemic, composed of physicians from preventive medicine, infectious diseases, the microbiology service, occupational hazards, medical management, and the hospital management. The professionals emphasized that they sometimes had to draw on the experience of colleagues from other centers.

“We had no contact with the patient, but there was contact with the coordination, authorisation, review of guidelines. We did not know what we were facing, … in the first wave we had doctors, but they were not well deployed, there was no organisation to help those who were overwhelmed, we had no one to do PCRs, there was a lack of information, resources of all kinds … someone would say I have a friend in the other hospital who is doing it this way. Let’s give it a go. […]”

Polymerase Chain Reaction, diagnostic test that detects a fragment of the genetic material of a pathogen.

The first three actions in Emergency Services were: the relocation of healthcare professionals based on safety criteria and risk profiles; the setting up of separate incoming and outgoing care circuits to avoid contact with non-COVID patients, and the organization of a “nursing kit,” which consisted of prescribing medication in a single visit, and coordinating with colleagues to minimize contact with the patient.

“At the beginning of March we had already established two care circuits, one for respiratory patients and one for non-respiratory patients. … and we did a little bit of distribution of professionals […]. We made kits for … nursing …, for when we had to [assist] a patient with everything: with compressors, with IVs, with tubes for blood tests, trying to expose the patient as infrequently as possible.”

During the second wave, the Internal Medicine Service set up the Intermediate Respiratory Care Unit (IRCU) with the aim of reducing admissions to the ICU by acting as a retention filter to reduce admissions to the Intensive Care Units. On-call duty was reinforced, and specific units were created with auxiliary/support teams made up of healthcare professionals with no direct link to the disease, thus extending the traditional work teams and strengthening the much-needed spirit of collaboration and synergy between specialties.

“…in the hospital, in the third wave, the unit that we set up in intermediate respiratory care. … was a success, because we were able to get a lot of people through it without their having to go to the ICU.”

“Being able to collaborate between specialties was very important, […] specialties such as rehabilitation, anaesthesia, neurology, psychiatry … the care teams were greatly expanded; in addition to nursing, which has a very important role, especially in critical care, there was a spirit of collaboration and synergy that helped a lot.”

In the Intensive Care Unit, due to the reduced pressure of care in the first wave, they were able to focus on the procurement of protective equipment and hospital beds. The training of other healthcare professionals was carried out informally, sharing knowledge focused on “day-to-day” experiences. This collaboration was highly appreciated by the healthcare professionals of this service, as both parties were more receptive and motivated.

“The first wave was quite gentle, the difficult part came later, then what we did was to prepare beds, material, purchases, just in case, […] in the first wave professionals from other specialties came to train in the ICU, people were more receptive, they were not tired … people were up for it; I think that was the difference in a way, to start strong, you were going into the unknown, but you knew you had to be there, it had to be done.”

### 3.4. Impacts of the pandemic on care provision

From the start of the pandemic, routine health services were reorganized or disrupted, and this undoubtedly had an impact on the delivery of care, which was a challenge for staff. Maximizing the number of patients who got through the disease was the biggest challenge and one of the most satisfying successes described by the healthcare professionals interviewed. Internists highlighted the challenge of finding effective treatments, showing great concern when patients did not respond to treatment. They also pointed out how they managed high-stress situations appropriately, given the limited resources available.

“What worried me most was not being able to offer patients the quality of care they needed, not being able to diagnose them in time, treating them with drugs that you often knew were ineffective; after 20 years of experience in the field of infectious diseases, the biggest challenge for me was looking for effective treatment alternatives …”

During the first wave, one aspect highlighted was the low level of infection among health workers in the workplace, despite the ignorance around preventive measures to curb transmission of the disease. Moreover, HHU professionals pointed out that working in an out-of-hospital environment facilitated the arrival of protective material from private sources.

“We spent the whole of the first wave without masks. Masks were stipulated at the beginning when you were with a patient who had a cough and was ill. And now that we know that air is one of the means of transmission … Well, we’ve been lucky …! Here in the department very few of us have had it, very few.”

“Honestly, we managed because of outside help … They phoned us to say, do you need us to get you masks? … And we got them from the relatives of the patients, how is it that relatives can get them and the management and the hospital can’t procure them for me?!”

Over time, during the second and third waves, the quality of care has been referred to as one of the main concerns for Internal Medicine Service, ICUs, ERs, and professionals in management roles, with the lack of physical space to accommodate patients being one of the major challenges. In the Emergency Service, this problem occurred earlier, as it is the gateway to the hospital care circuit. Also, the nursing staff of the HHU underlined the lack of time for proper care of non-COVID patients, especially palliative care patients and their families.

“The challenge in the ward was trying to organise everyone coming in, the incoming avalanche, we had nowhere to admit those patients!”

“Being unable to devote time to our palliative patients. […] We try to provide support over the phone and in a hurry … that made me think: ‘I’m not doing my job properly.”

In the Intensive Care Unit, the lack of specialized training in treating critically ill patients was a constant problem.

“ICU staff are trained in this, but the rest of us? It’s like being thrown headlong into something I know nothing about.”

Two further sources of problems are described: (1) the new incorporations to Intensive Care, mainly of Resuscitation and Anesthesia professionals, which led to conflicts, due to the continuous demand for help by these professionals from ICU professionals overwhelmed by the workload they were facing; and (2) the contents of the training courses on offer, which are described as very basic, and not focused on the professional profile required.

“As the waves became longer and bigger, all kinds of professionals came […] they couldn’t be trained because we couldn’t keep up, they came to work, but in the wards straight away, because there wasn’t enough time. It was a bit chaotic.”

“And then we had to work outside the ICU with anaesthetists in their spaces, in operating theatres, in rehabilitation. And there the relationship was more difficult, because they, the anaesthetists, demanded, quote unquote, much more support … for us to be constantly at their side. […] But, what we faced was so unmanageable that it was impossible, we just couldn’t cope!”

“In the second and third waves, there were some training courses … lots of people signed up, but in the end, these were not the people for whom the course was intended …, the course was very basic for people in the Emergency Service, ward doctors who came to be trained were few and far between. This means that there have been patients who have been treated differently, and who have had different outcome chances, […] in the end it was the patient who lost out.”

Coordination between Internal Medicine, Emergency, ICU, and other support services was essential. This required standardization of all procedures, treatments, and preventive measures to facilitate the adaptation of external professionals. Teamwork was a key factor in improving quality of care results.

“Everything had to be done by the book, which was updated on a daily basis. We tried to standardise everything.”

“The success … was the result of everyone’s work; day after day, shift after shift, colleague after colleague …, for a very long time […] you’ll have seen it on TV, when the patients were being discharged from the ICU, how everyone joined them … you can’t imagine the amount of work and the number of hours, and the amount of effort, that lay behind it all …!”

The only Primary Care healthcare professional in the sample reported his helplessness and even the occasional clash with colleagues when he offered in-person assistance at the health center, instead of by phone, in breach the service’s guidelines.

“Dealing with someone on the phone and trying to guess if they’re unwell, is very complicated. You do things that you wouldn’t be … quite comfortable with in other situations. […] We had express orders that, if you had a Covid patient and they were unwell, then straight to the hospital without seeing him. I can’t do that. […] Patients don’t like it either… I’ve had the odd clash with colleagues along the lines of ‘Never mind what happens, I want to explore further’ […] because the orders that came from above were different from what many of us wanted to do.”

### 3.5. Impacts of the pandemic on the health of healthcare workers

The major psychological impact reported was due to the increase in deaths, the application of “war triage” in the selection of patients, and the fall in the age profile of COVID patients. All this was in a context of scarce material/human resources and restrictive rules that prevented face-to-face communication with relatives.

“The biggest impact … above all, we weren’t used to seeing so many people die, because when the ICUs became very busy with beds, a war triage was applied. […] A limit was set for entry to the ICU, because you had to keep the beds for the younger people who were going to arrive, … those older patients stayed on the ward, they were the ones we saw and we had to try to get them through with the means we had, […] people couldn’t have visitors, they died in the company of the health staff, without their family around them, that was also hard and complicated.”

All the healthcare workers reported emotional stress at some point during the period under study. In the first wave, situations of anxiety and worry are described. In the third wave, events are described as being accompanied by distress and high stress, the main health problems being: hypertension, hair loss, headaches, back pain, and insomnia.

“It was mostly anxiety, a lot of nervousness. Insomnia from time to time.”

“I was unwell, I had backache, headaches … There were moments of anxiety, of stress, maybe of bad language, I think that … we were all almost the same …, because we were all very tense.”

“I had a hypertensive crisis … My hair fell out. People are exhausted and worn out.”

Despite this, no healthcare professional took sick leave due to the situation of stress or overwork; adapting to the intense workload was the standard coping method, leading to a period of mechanical work which became gradually routine. Moments of de-escalation or deceleration allowed physical exhaustion to manifest itself, especially persistent psychological fatigue. During these periods, the dialogs once again reveal a sense of uncertainty as to the future evolution of events.

“I think there has been a very great deal of work and we have had to address very complicated situations, and I personally have not missed a single day of work.”

“… when the plateau was reached, when no more patients were arriving, there was a sense of relief, but the tiredness was there.”

Differences in perceived stress were observed according to functions and services. Preventive Medicine healthcare workers acknowledge that they have “*suffered*” less pressure than colleagues on the front line (ICU, Emergency, and Internal Medicine Services), who spoke in the third person to highlight that it entailed a collective collapse rather than that of individuals.

“I have been fortunate enough not to be with patients, but I have suffered.”

“As for very tired colleagues, they said yes to everything, but always when saying yes, they added: ‘but we’re tired’. I understand, I understand, but we must carry on, come on, this is going to end.”

#### 3.5.1. Institutional sources of support, coping strategies, and feelings of support

The restructuring of work shifts was a source of institutional support that was seen as important and was highly valued. It was based on patient volume and staff safety, enabling rest breaks during de-escalation. Shared shifts with healthcare professionals in the same specialty also reduced the emotional burden of sharing knowledge and responsibilities in therapeutic decision-making. “Bubble groups” were created to reduce interactions and the risk of transmission. However, this measure was insufficient, as staff shortages have been a constant obstacle to better results.

“We went from doing our 8-hour shifts plus on-call duty to 12-hour shifts. You’d spend the whole day at the hospital. We practically lived there, and this was done precisely so that we didn’t coincide with others, to make bubble groups.”

At institutional level, teamwork was encouraged, helping to foster solidarity among “colleagues” as a collective coping strategy, reducing the workload, enhancing feelings of loyalty, security, self-esteem, and enabling coordination-cooperation in the tasks to be performed. Indeed, these informal support networks, in the work context, have fostered friendly relationships and camaraderie, especially among nursing staff, and the expression “hacer una piña” (“becoming a pine cone,” meaning all pulling together) was often used in this context.

“For me, fortunately, we are a team, we were eight nurses and six doctors. We all pulled together [hicimos piña], especially the nursing staff, and we protected one other. One colleague would take on more work so that two of us could leave together, to help each other get dressed and undressed … among colleagues we did group therapy, we tried to take positives from the day, … always looking at the funny side […] in bad times we supported each other and we helped each other a lot.”

The psychological counseling for healthcare workers introduced at the end of the third wave was valued positively, especially by the Intensive Care Unit staff; however, they point out its limited effectiveness and use, due to time constraints, and to its not having been implemented from the beginning. Management nursing staff had to offer this support to the members of their team, and consider it part of their daily routine.

“A psychologist was hired by the hospital towards the end and there were mindfulness sessions, all late in the day to my mind, but anyway, it was done, though I personally didn’t go to any.”

“what I saw was that morale was … I was there sort of to help everyone! My office was open, I had an hour of psychology, they went in and cried, I had to listen to them, because it was part of the job, until the psychologist came, so I did what I could.”

At the same time, social support strategies developed in the private/personal context were mentioned frequently by all personnel. Sports activities were highlighted by more men than women as a means of escape and distraction in times of stress, especially in the third wave.

“Well, I relied on my family, of course … when I got home, when my husband and my son were at home, […] which might be 9 pm, I would sit down with them, have a beer, and talk.”

“Well, I channel it all pretty much through sport. That does it for me.”

In general, the use of medication was rarely mentioned as a regular source of relief, and there was some reluctance when it came to communicating this, suggesting contradictions and even rejection; just three women explicitly mentioned the use of medication at times of increased stress. Insomnia was one of the problems that gave rise to the prescription of medication.

“Well, since the first wave, it’s true that I was on medication to sleep at first, for the anxiety and the pace we were going at, I couldn’t sleep at home.”

“No, no, no, no, no medication, but I did take valerian, camomile. But, hey, not sleeping pills or benzodiazepines, at least at the beginning. I prescribed them for myself in April, but in the third wave I didn’t need them […] we’ve talked about it, there have been colleagues, yes, who have self-medicated, to get to sleep and to feel calmer…”

In general, healthcare professionals in management roles felt institutionally supported, although there are contradictions in what they say.

“What we asked for was granted, you always have to fight for it a little, but well, I felt that they supported me […] to fight for it in the sense that it was part and parcel of the functioning of the health system.”

However, healthcare workers with direct care roles, mainly ICU, Emergency, and Internal Medicine staff, have felt more unprotected, unheard, and neglected by the hospital management. The overload and fatigue have been overcome by the high levels of vocational commitment and professional dedication they have shown and the challenge of “saving as many lives as possible” from the very beginning. The search for support outside the hospital environments was a solution that most healthcare workers saw as the only effective option at that time.

“I think the management should have had a little more dialogue with us. What do you need? You need something, but nothing was forthcoming. In other words, what you can’t do is ask the question and not provide. […] And then they want you to go back to normal and go back to work, as if nothing had happened […] undervalued or not appreciated by management, who left us to our own devices, you do a job and on top of that they don’t thank you for it. That’s about the size of it. That’s how it feels […] We were used when we were needed and then goodbye.”

“My husband said to me, why don’t you take a leave of absence? I CAN’T! I need to work, because I was overwhelmed, but at the same time I felt fulfilled to be helping all those people [patients] […] those at the top don’t care about how you feel. You have to look for … your relaxation techniques, your own way.”

ICU, Emergency, and Internal Medicine staff have described, more than others, emotional control coping methods, such as: relaxation techniques, emotional distancing, apathetic behavior, and acceptance.

“… some people tell me I’m uncaring, if you’re not uncaring in the ICU, you die of grief within a week…!”

“I saw from the first minute that people were going to die because I wasn’t going to take care of them as one would a sick person in other situations. […] But in general, on a personal level, I handled it well, I was very, very cold in that aspect, and so I managed to be efficient.”

#### 3.5.2. Recovery levels

Feelings of frustration, weariness, exhaustion, and fear are still present in all the healthcare workers interviewed, so the need for rest is repeatedly highlighted. More women than men (4 vs. 1) have explicitly noted a failure to recover their health. Also, the reference exclusively to the professional environment is unanimous among the men (4 vs. 1) who claimed complete recovery. In fact, the only woman who said that she had recovered had some doubts as to the psychological consequences that this experience may have left her with.

“I don’t think I’ve recovered, there’s still something there, because I haven’t recovered, because what I do think is that I’m afraid, although I don’t say so (laughter).”

“The doubt that stays with me is whether it has left me with some kind of after-effects … on a psychological level …”

### 3.6. Proposals for improvement

An increase in trained personnel to avoid work overload and to facilitate rest has been the measure, broadly, most frequently mentioned by all healthcare workers to avoid a repeat situation should the pandemic re-emerge.

Staff of the Preventive Medicine Services have suggested not dismissing trained personnel, echoing the collective feeling. They referred to the need for a contingency plan for future pandemics with further training of existing personnel to address the lack of human resources in these specialties.

“I think there should be contingency plans and think that, if we need more intensive care staff and they’re not there, maybe the anaesthetists need to be trained in this type of patients.”

The Intensive Care Unit pointed out the need for training in pandemics and the importance of continuous training. They particularly underlined the need for specialized training in critical care for staff at all levels who join the service.

“What I would improve …, the most vital thing, is that people are trained, that they’re people with experience, people who’ve worked in an ICU, people who don’t need supervising or teaching. […] we’re looking into the issue of the specialty of critical care nursing, which is still not recognised, it would be good…”

Most professionals agree that psychological help should be established as a regular resource of special importance. Despite its implementation, they were pessimistic about it being retained.

“In the critical care unit there should always be a psychologist because … it would help us to talk to each other more, to communicate, because communication is a bit so-so in the ICUs, you don’t need to laugh every day.”

“The psychologist has been a great help for the last two months, and if she’d been there before, she could have helped a lot more. I’m afraid that when the pandemic is over, her contract will expire.”

The Internal Medicine Service proposes avoiding the excessive moving around of work teams, which made it difficult to establish stable work teams with the requisite training.

“I’d have left the same people on the same floor, not rotating; one minute I’m going to the 3rd floor…, tomorrow, we’ll see …”

This last discourse identifies both criticism and areas for improvement by assessing the elements that have failed not only from the point of view of hospital organization, but also at national and international level. This section highlights the lack of foresight of human and material resources, the lack of assertiveness of WHO recommendations, the confusing and unsubstantiated messages in the media, and finally the lack of social awareness of universal responsibility to adhere to the restrictive rules put forward by the WHO.

“Aspects to improve, foresight […] the WHO warnings weren’t taken seriously, because the WHO didn’t believe in them either [laughs]. The technicians believed them, but the politicians didn’t believe them. I think the WHO should have been much more assertive in its recommendations. The countries that closed early in the first wave were less impacted and other countries like Spain [weren’t] … you just knew what was coming! The logistics parameters also failed. The media failed, I think they didn’t offer a clear message; in other countries they were more in your face, the corpses, inside the hospitals, the care homes, the people who lived on the streets, the extent of the economic impact, it’s all very well, but doesn’t help. […] And then another area that undoubtedly needs improvement relates to the awareness levels of many people. We have seen infected people who have not followed quarantine. It’s true that if we had a legislative system that allowed these people to be sanctioned, to be properly quarantined, a large proportion of cases would have been avoided.”

## 4. Discussion

This study offers important findings about the experiences of healthcare professionals based on their service and role during the first three waves of the COVID-19 pandemic. First, the news was received very differently by different services, in some with disbelief, and in others as an opportunity for professional development and job improvement. The lack of leadership and widespread misinformation during the first wave generated feelings of helplessness, uncertainty, and fear relating to job security, contagion, and/or transmission to family members. Even so, the initial response saw organizational changes often driven by the services themselves, which in itself proved very positive, although in some cases limited by scarce resources (material and human). The quality of care throughout the three waves was a constant concern of these healthcare professionals, mainly due to the lack of effective treatments, of sufficient physical space, of adequate training in treating critical patients, and to the lack of human resources together with a lot of moving from one area to another. Despite this, in the third wave, which brought the greatest overload in our sector, the health problems of the healthcare workers did not lead to sick leave; we believe that the high level of commitment and vocation observed were factors that helped people adjust to the intense work patterns. We did observe a growing psychological fatigue that became chronic due to the perception of being faced with a long process with no end in sight.

At an institutional level, the organization of work shifts facilitated rest and reduced overload, although the psychological help offered was not useful, as it was late in coming. The coping strategies referred to were varied, with the following standing out: family support, support from work colleagues, and sporting activities. It is worth highlighting the greater use of emotional control techniques by ICU, Emergency, and Internal Medicine staff, who, after expressing higher levels of stress and feeling “poorly supported” by hospital management, opted to seek the help they needed outside the hospital environment. At the time of the interviews, healthcare staff noted that they were in the process of recovery, albeit with negative feelings and a lot of fatigue.

Other studies have found similar results to ours: constant changes in guidelines, lack of institutional foresight, lack of clear information, prolonged use of PPE, fear of infection, and infecting family members all adversely affected an adequate response, causing uncertainty and increasing the stress of healthcare professionals (19–23).

The adaptive capacity of the health system has been one of the factors that determined the greater or lesser success of the response to the COVID-19 pandemic (6). In Spain, the ENCOVUR study of the organizational impact on the management of the pandemic noted that, during the first wave, one of the main problems in caring for COVID patients was the lack of adequate physical space, more frequent in small- or medium-sized hospitals, and in those in areas with higher incidence levels (24). A study carried out in 31 European countries indicates that during the first wave, there was an inefficient response by health systems in most western countries, including Spain. Additionally, the complexity of the COVID patient and the lack of specific treatments worsened the quality of care (25). Subsequently, in the relaxation phase, those states that were severely affected at the outset began to take appropriate measures and improve the efficiency of their health systems (26).

A systematic review of 40 studies mostly conducted in the USA and the UK highlighted that the best training for healthcare professionals in the treatment of COVID patients was gleaned from more experienced workers (27). In our study, this was hampered by its low incidence during the first wave, and in the following waves by the high turnover of healthcare staff.

Professional vocation, inner strength, and peer support are also cited in numerous studies as a source of motivation and adaptation in responding to intense, unrelenting working hours (28–32).

Institutional and psychological support has also been referred to in other studies. In one study, nurses “took the initiative to be altruistic and sought team support” as a coping strategy (22). Strong institutional guidance and committed leadership were positive initiatives for mental health described in a study conducted in 13 countries in different healthcare settings, including Spain (33). In another study, psychological support relieved nurses’ anxiety. This support was even more appreciated when it was adapted to shift work, was informal, and based on individual or small group dynamics (34). However, in some Chinese hospitals where a detailed psychological intervention plan consisting of counseling courses, individual supervision, and group interventions was developed, doctors were reluctant to participate and nurses rejected it, most of them arguing that they did not need a psychologist, but rather a break and sufficient protective equipment (35).

Family, social, institutional, and peer support to reduce the negative impact on healthcare workers’ health during the pandemic was significantly associated with individual performance, sleep quality, motivation, and better mental health (32, 36, 37). However, perceptions of institutional support have varied across countries: Whereas in China (38) and/or Jordan (37), healthcare professionals reported confidence in their organization, in North America (39), the Mental Health America (MHA) survey concluded that 39% of health professionals in the United States did not feel emotionally supported, with the level for nurses being as high as 45%. Family and friends accounted for more than half the support received, followed by work colleagues with 38%, while supervisors and therapists accounted for around 15%.

The perception of greater psychological distress in more caring roles has also been described (40–42). In Spain, a higher prevalence of PTSD was found in front-line healthcare workers (43), especially in nurses (10). Finally, the results of the present study may be constrained by the lack of face-to-face interaction between interviewers and interviewees, which restricts information from non-verbal communication and context observation for the researcher. Nevertheless, we believe that our results provide a rich insight into the experiences of healthcare professionals from different services.

## 5. Conclusion

Despite the high levels of emotional stress reported, no sick leave was taken. Health professionals showed a strong collective spirit and sense of solidarity. Family, social support, and camaraderie at work were effective coping strategies. The direct experience of professionals suggests the need for a contingency plan adapted to each organizational context, which includes psychological support, the provision of adequate materials and effective organizational measures learned during this pandemic. Proposals for improvement include the need for continuous training in critical patient care at all levels of care, as well as the use of existing staff who gained training and experience during this pandemic.

## Data availability statement

The original contributions presented in the study are included in the article/Supplementary material, further inquiries can be directed to the corresponding author.

## Ethics statement

The research was approved by the ethical committee of the hospital. However, due to a confidentiality agreement between the participants and the research team, the name of the hospital cannot be disclosed. The participants provided their written informed consent to participate in this study. Written informed consent was obtained from the individual(s) for the publication of any potentially identifiable images or data included in this article.

## Author contributions

MP-V, EA-L, and GP-M conceptualized and designed the study. MNR-M conducted the semi-interviews. MNR-M and MP-V carried out data analysis. MNR-M produced a first draft of the results section and wrote the first draft of the document that was extensively refined by all authors until its final version. All authors contributed to the article and approved the submitted version.

## Funding

The field work and the article-processing charge was funded by the Center for Biomedical Research Network (CIBER), Carlos III Health Institute (ISCIII); your help is greatly appreciated.

## Conflict of interest

The authors declare that the research was conducted in the absence of any commercial or financial relationships that could be construed as a potential conflict of interest.

The reviewer AM declared a past co-authorship with the author MP-V to the handling editor.

## Publisher’s note

All claims expressed in this article are solely those of the authors and do not necessarily represent those of their affiliated organizations, or those of the publisher, the editors and the reviewers. Any product that may be evaluated in this article, or claim that may be made by its manufacturer, is not guaranteed or endorsed by the publisher.
