# Supplementary material for: “You knew you had to be there, it had to be done”: Experiences of health professionals who faced the COVID-19 pandemic in one public hospital in Spain
Source: Front Public Health. 2023 Apr 20;11:1089565. doi: 10.3389/fpubh.2023.1089565 (PMC10170551; doi:10.3389/fpubh.2023.1089565)
Supplement: Supplementary file 1 [file Table_1.docx]

Appendix 1. Semi-Structured Interview

| **Motivation and professional vocation** |
| --- |
| - What led you to take up this profession?  - Do you feel fulfilled by it? |
| **Care provision and pandemic management** |
| - How did you receive the news of the pandemic?  - What was your reaction?  - How did you feel the first day?  - What were the main differences in your usual work performance before and during the first wave?  - What prior training/information did you have on health care in pandemic situations?  - How do you feel now?  Next, think about your day-to-day experience during these 3 waves:  - What everyday situations had the greatest impact on you?  - What were the main concerns during this period?  - Tell us about a challenging professional experience you faced during this period. Also an experience that has been a success for you. |
| **State of health** |
| Did you catch the disease?  or If you did, how did you manage the situation?  or If you did not catch it, were you afraid of catching it? Were you afraid of infecting those close to you?  - Do you believe that working in a hospital during the COVID-19 pandemic has had an impact on your health?  - What kind of symptoms did you experience or are you experiencing?  - Do you think you reached the point of breakdown/emotional overload?  - Could you identify the factors or circumstances that caused you the greatest levels of emotional distress?  - Did you require assistance of any kind, if so what kind, did you request it at this stage?  or If you requested it, was it easy to obtain?  - Did you require treatment of any kind at this stage? What type of treatment? |
| **Sources of support at institutional level** |
| - Did you feel supported by the hospital management? - Did you feel that the hospital organisation emphasised self-care for healthcare professionals? - Was there continuous training and education of health professionals in response to new scientific evidence as it became available? If the answer is yes, then: - What kind of support did you receive from your hospital during this period? - Was the support helpful? - How did it help you? - How was the relationship with your co-workers? - Did you have to work with professionals from other services who though it was not their specialty worked alongside you in the treatment of COVID patients, was that situation stressful, or did you manage to function well as a team and maintain the quality of care? - In your view, what elements facilitated or would have facilitated the management of stress and burnout situations for healthcare workers in your specialty? - If you could implement preventive measures in the hospital to lessen the psychological impact of a situation like COVID 19, which would be the most effective in your view? - And of these measures you mention, which do you consider the most important? |
| **Self-management and coping strategies** |
| - Could you explain what your personal coping strategies were for dealing with breakdown/emotional overload? - To what extent have you recovered or do you feel that you are still suffering from the physical or psychological effects of the pandemic? - Do you think this experience has improved you as a professional? |
| **Responsibility for the provision of care** |
| - Are you satisfied with the work you have done from the beginning of the pandemic until now? - Tell us about the positive and negative aspects of your work during this period. - Over the last year, which aspects of your work have improved, got worse and remained unchanged?   To conclude:   - What did you think of the interview? - Do you have any suggestions for improvement, what are they? - Is there anything about this subject that I have not asked you and that you would like to tell me? |
| THE END |
